# Supplementary material for: The P2X7 Receptor Promotes Colorectal Inflammation and Tumorigenesis by Modulating Gut Microbiota and the Inflammasome
Source: Int J Mol Sci. 2022 Apr 21;23(9):4616. doi: 10.3390/ijms23094616 (PMC9099551; doi:10.3390/ijms23094616)
Supplement: Supplementary file 1 [file ijms-23-04616-s001.zip › ijms-1678742-supplementary.pdf]

Supplementary Results:

Supplementary Figure S1

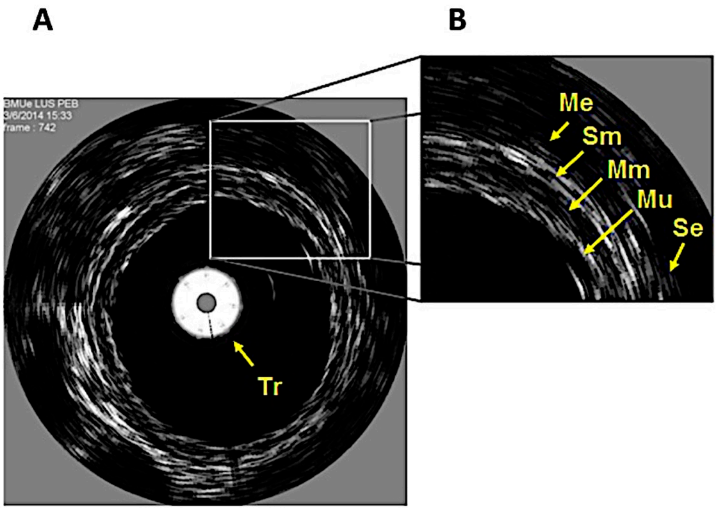

Supplementary Figure S1. Endoluminal ultrasound biomicroscopic (eUBM) imaging of the colon. The eUBM system was used through video colonoscopy to detect and characterize lesions simulating human IBD and colitis-associated colorectal cancer in the colon of mice in vivo. The example shows details of the intestinal layers. The more echogenic (hyperechoic) layers produce the lightest images, and the least echogenic (hypoechoic) layers produce the darkest images. **Tr**, ultrasound transducer; **Me**, muscular layer, outer muscle (hypoechoic layer); **Sm**, submucosa (hyperechoic layer); **Mm**, muscularis mucosa (hypoechoic layer); **Mu**, mucosa (hyperechoic layer); **Se**, serosa (hyperechoic layer).

Supplementary Figure S2

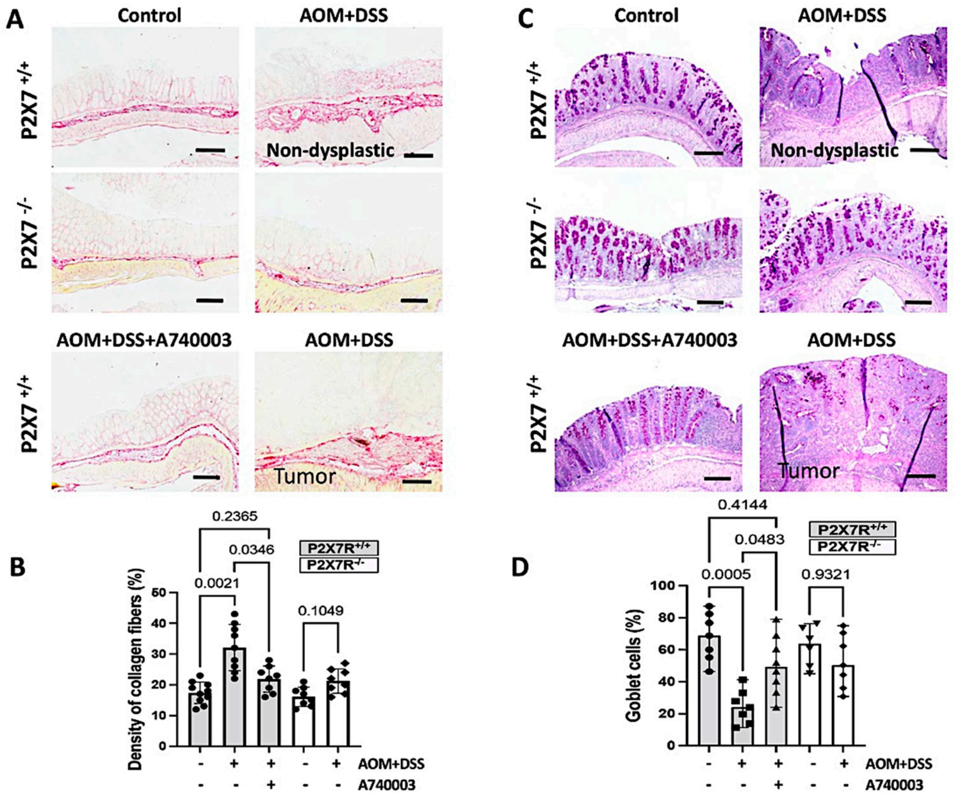

Supplementary Figure S2. P2X7R blockade attenuates fibrosis and epithelial damage in the AOM/DSS-induced mice. The P2X7R$^{-/-}$ and P2X7R$^{+/+}$ mice treated with the P2X7R antagonist A740003 did not accumulate collagen fibers labeled with picrosirius red dye (A, B) and lost less differentiated mucous-producing goblet cells labeled with periodic acid-Schiff (PAS) (C, D). Values are the medians with interquartile ranges of three independent experiments, with 3 to 4 animals per group. The scale bars represent 20 µm. The analysis was performed by Brown-Forsythe and Welch ANOVA tests, in which multiple comparisons were carried out using Dunnett's T3 test. Significant values are presented.

Supplementary Figure S3

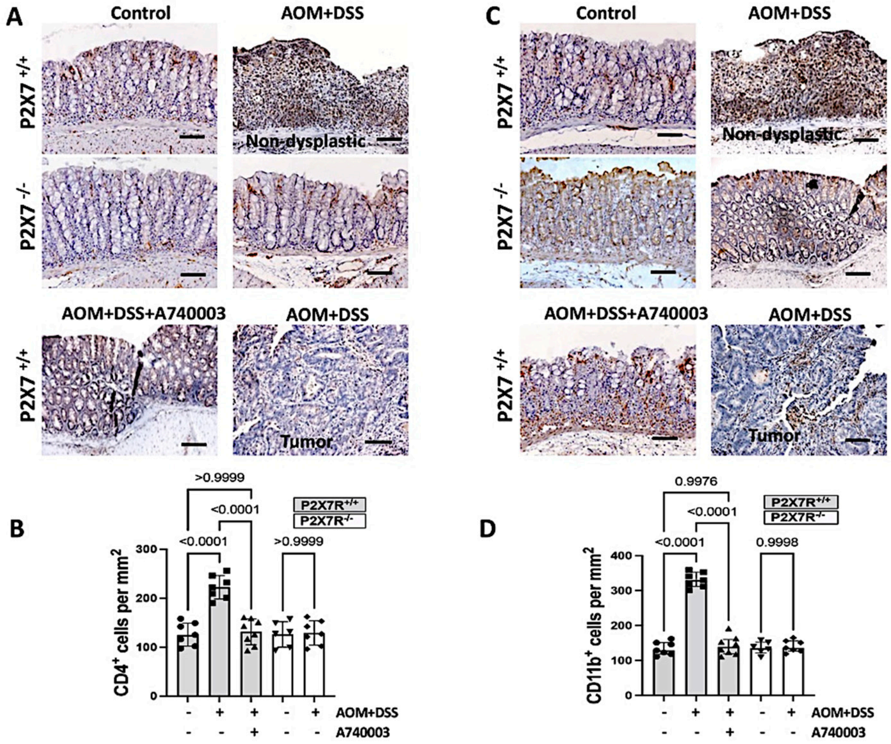

Supplementary Figure S3. Nondysplastic inflamed areas of the colon of the AOM/DSS-induced mice are characterized by intense inflammatory cell infiltration in the lamina propria, including CD4- and CD11b-positive cells. The P2X7R$^{-/-}$ and P2X7R$^{+/+}$ mice treated with the P2X7R antagonist A740003 did not accumulate CD4- (A) or CD11b-positive cells (B). Values are the medians with interquartile ranges of three independent experiments, with 4 to 5 animals per group. Significant values are presented. The scale bars represent 20 µm. The analysis was performed by Brown-Forsythe and Welch ANOVA tests, in which multiple comparisons were carried out using Dunnett's T3 test. Significant values are presented.

Supplementary Figure S4

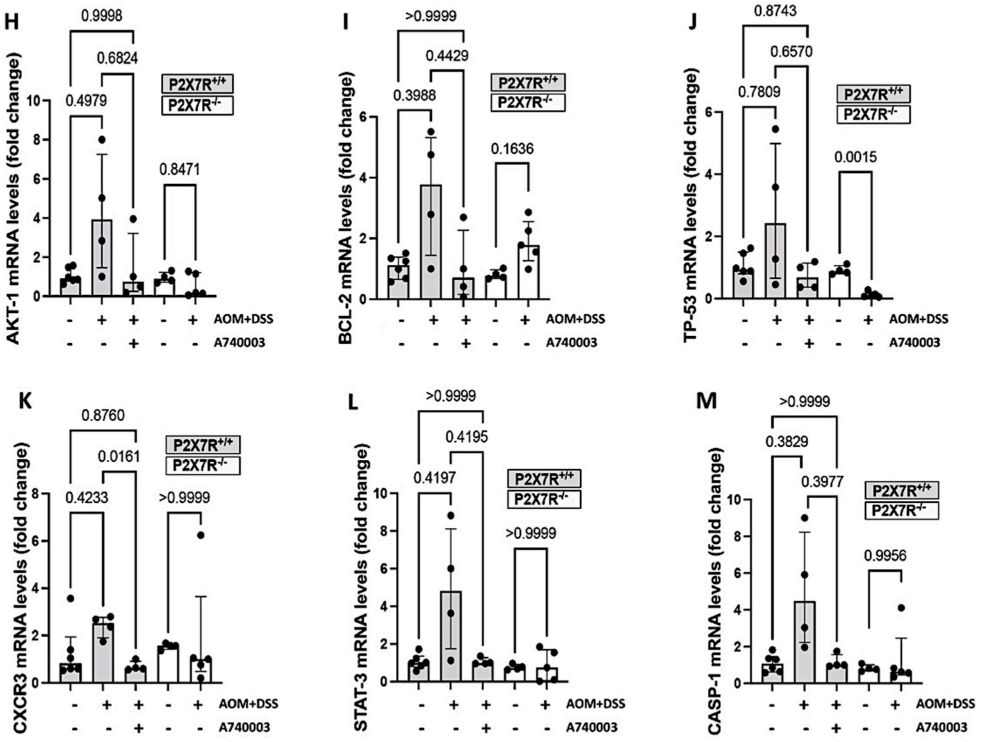

Supplementary Figure S4. Effect of P2X7R blockade on the expression of genes related to inflammation and colon cancer in the AOM/DSS model. mRNA was measured by quantitative real-time PCR in colon samples. Although the mRNA levels of the *AKT-1* (A), *BCL-2* (B), *TP-53* (C), *CXCR3* (D), *STAT-3* (E), and *CASP-1* (F) genes tended to be higher in the AOM/DSS-induced P2X7R$^{+/+}$ mice, no significant changes were detected among the experimental groups. Values are the medians with interquartile ranges of three independent experiments, with 3 to 4 animals per group. The analysis was performed by Brown-Forsythe and Welch ANOVA tests, in which multiple comparisons were carried out using Dunnett's T3 test. All statistical values are presented.

Supplementary Figure S5

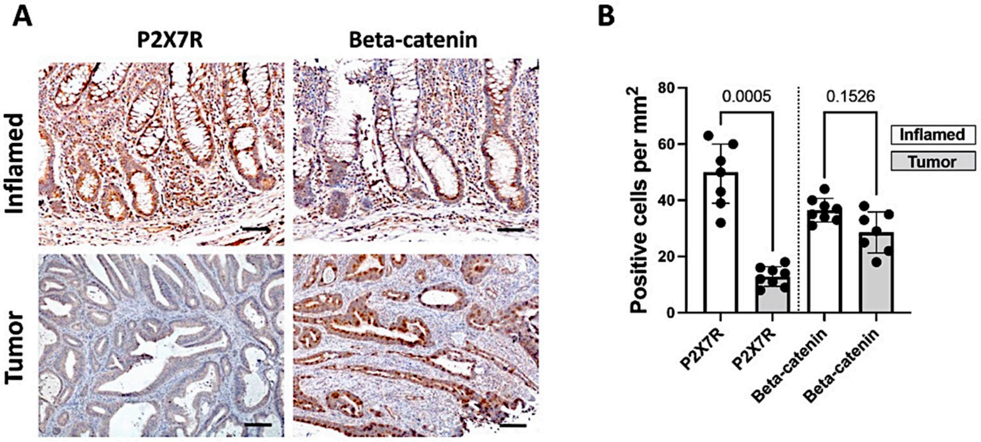

Supplementary Figure S5. P2X7R and beta-catenin differential expression in tumors and inflamed areas of the colon. Immunoperoxidase experiments revealed diffuse staining of P2X7R in the lamina propria and epithelial cells of the inflamed areas of the colon, while in the tumors, only faint staining was observed in both compartments. Beta-catenin staining showed a similar distribution compared to P2X7R in the inflamed areas, but in the tumors, the expression was predominantly epithelial, with strong staining clearly concentrated in the cell nuclei. Values are the medians with interquartile ranges of three independent experiments, with 3 to 4 animals per group. The scale bars represent 20 µm. The analysis was performed by the Wilcoxon matched-pairs signed-rank test. Significant values are presented.

Supplementary Figure S6

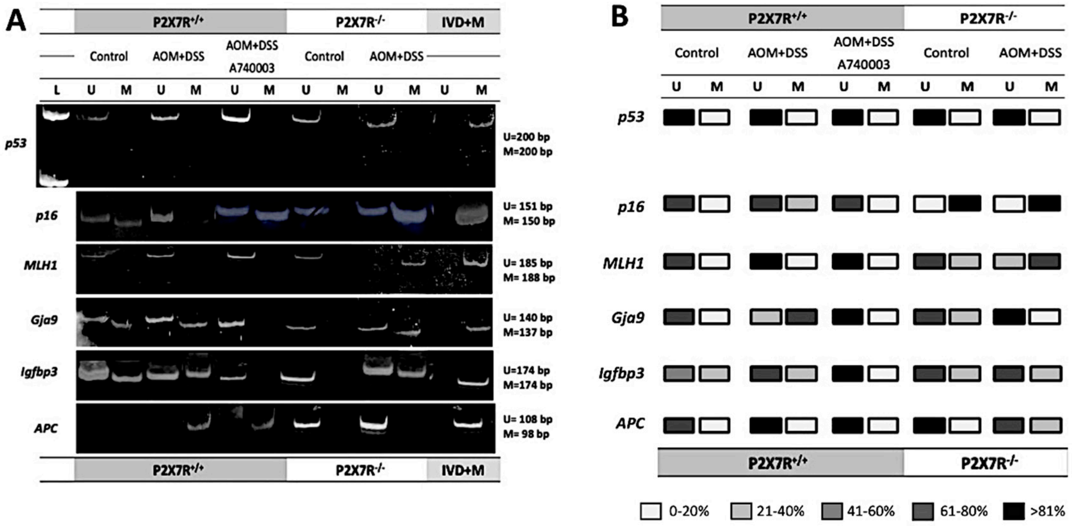

Supplementary Figure S6. Effect of P2X7R blockade on the methylation pattern of genes related to inflammation and colon cancer in the AOM/DSS model. Methylation-specific PCR gel examples for the *p53*, *p16*, *MHL1*, *Gja9*, *Igfbp3*, and *APC* promoters were analyzed. Methylated (M) and unmethylated (U) genes are indicated (A). Methylation profiles and percentages are depicted using a grayscale, with a white box representing unmethylated promoters and black representing methylated promoters (B). In vitro DNA (IVD) was used as a positive control for methylation, and a DNA ladder (L) is indicated. Data are representative of three independent experiments, with 3 to 4 animals per group.

Supplementary Figure S7

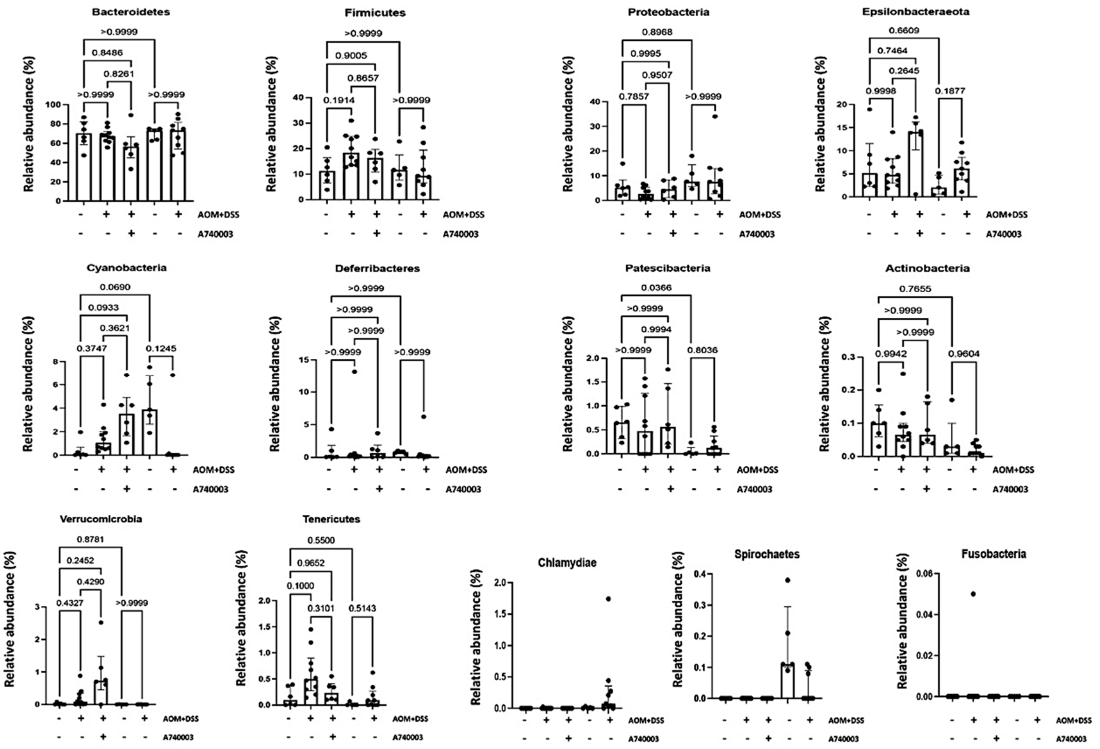

Supplementary Figure S7. Effect of P2X7R blockade on microbiota composition in fecal samples in the AOM/DSS model. The relative abundance of operational taxonomic units in bacterial phyla is presented (%). No significant changes were detected among the experimental groups. Values are the medians with interquartile ranges of 5 to 10 animals per group. The analysis was performed by Brown-Forsythe and Welch ANOVA tests, in which multiple comparisons were carried out using Dunnett’s T3 test. All statistical values are presented.

Supplementary Figure S8

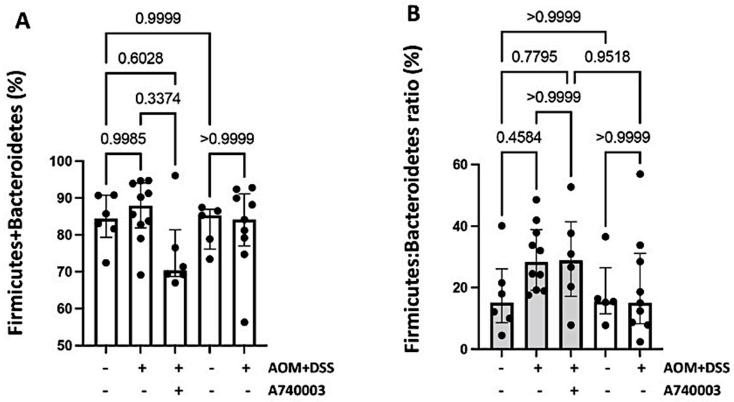

Supplementary Figure S8. Effect of P2X7R blockade on microbiota composition considering the Firmicutes + Bacteroidetes (A) and Firmicutes: Bacteroidetes ratio (B) of fecal samples in the AOM/DSS model. No significant changes were detected among the experimental groups. Values are the medians with interquartile ranges of 5 to 10 animals per group. The analysis was performed by Brown-Forsythe and Welch ANOVA tests, in which multiple comparisons were carried out using Dunnett's T3 test. All statistical values are presented.
